# Validation of the Index for Inclusion Questionnaire for Parents of Non-University Education Students

**DOI:** 10.3390/ijerph17093216

**Published:** 2020-05-06

**Authors:** José A. Fernández-Archilla, José M. Aguilar-Parra, Joaquín F. Álvarez-Hernández, Antonio Luque de la Rosa, Gerardo Echeita, Rubén Trigueros

**Affiliations:** 1 Department of Psychology, Hum-878 Research Team, Health Research Centre, University of Almería, 04120 Almería, Spain; archijaf@gmail.com (J.A.F.-A.); jalvarez@ual.es (J.F.Á.-H.); 2 Department of Education, University of Almería, 04120 Almería, Spain; 3 Developmental and Educational Psychology Department, Faculty of Psychology, Madrid Autonomous University, 28049 Madrid, Spain; gerardo.echeita@uam.es; 4 Department of Language and Education, University of Antonio de Nebrija, 28015 Madrid, Spain

**Keywords:** inclusive education, attention to diversity, families, validation, factorial analysis

## Abstract

The perspective from the parents of non-university students is essential in determining inclusive education in a school. The Index of Inclusion is one of the most widely used self-assessment tools and strategies to help teaching teams self-assess their political cultures and practices from the perspective of the values and principles of educational inclusion worldwide. For this reason, the present study intends to show evidence of validity of the Index for Inclusion questionnaire for parents of non-university education students, in a quantitative way, through a confirmatory factor analysis (CFA). In this study, 108 fathers and 500 mothers took part, aged between 21 and 62 years (*M* = 43.59; *SD* = 6.64), whose children belonged to educational institutions throughout Spain. The results revealed adequate adjustment rates, showing invariant structure with respect to sex. The Index for Inclusion for families of non-university education students was shown to be a robust and adequate psychometric instrument to assess the degree of development of inclusive education in educational institutions from the perspective of the parents of said student body. The family is a basic pillar in the education of children and a reference for them. In addition, parents of non-university education students are configured as fundamental participatory elements of the child’s educational institution thus; making the family a fundamental element that favors inclusive education. Precisely because of all this, the future administration of this questionnaire (to the parents of these students) is recommended.

## 1. Introduction

Today, one of the main priorities of educational institutions is to identify and respond to the diversity of needs of all students through increased participation in learning and reducing exclusion in education [1,2]. This definition of inclusive education (IE) demonstrates the importance of this type of process in the participation and commitment of all students in learning experiences [3], as well as in the relationships with their peers and adults, having favorable effects for the development of all children and adolescents [4,5,6].

On the other hand, Echeita [7] states that IE has the purpose of changing educational systems, so that all students, without exception, can fully develop their personality within the framework of a comprehensive and common educational system. In this way, educational institutions are composed of very diverse students, being a faithful reflection of today’s society. Responding optimally to the diversity of students in the educational and social environment has become an unavoidable issue in today’s educational breviary [8,9,10]. For this reason, Ainscow [11] advocates an education in which the discriminatory processes exhibited in behaviors and concrete responses towards diversity, caused, among other aspects, by reason of gender, ethnicity, social class, religion, sexual orientation, and disability, are suppressed. Thus, there is a desire to transform the school environment, improving it, an idea from which inclusion is proposed as an emblem, guaranteeing equity as well as quality [12]. In this sense, inclusion is configured as a challenge to which international education systems have joined to increase the quality of education and the response to the diversity of students, favoring learning opportunities for all, and estimating education as a liberating component that changes lives [13,14,15]. Moreover, as Ainscow, Dyson, Hopwood, and Thomson [16] assert, IE is not the sole responsibility of schools, but of society as a whole. Thus, from a new vision, a germinal posture of sending towards the active involvement of the local community, in which families play a fundamental role, in addition to volunteer networks, can be seen [12]. 

According to Porter and Towell [17], and continuing with the previous argument, we must admit that schools are a fundamental piece of society and that enriching education means working in coalition with students and their families. Therefore, complicity between families and the community in schools is considered a principle of educational quality [18]. Along these lines, as stated by Collet-Sabé, Besalú, Feu and Tort [19], an action plan based on the inclusion of all families should be implemented, focusing on the establishment of a good connection with them, and from a responsibility to combat the proliferation of social asymmetries, and for the academic achievement of all students. In addition, as Simón, Giné and Echeita [20] point out, families are an excellent way to support teachers in their work in the classroom and the school in the improvement processes. The tutors, parents, and other subjects in the family have information and approaches of enormous interest for the school to achieve the main goal of improving learning and the involvement of all students.

There has been an important development of standardized instruments aimed to support the transformation processes that education systems require on their way to an education, without exclusions, aimed at both teachers, students, and families. In particular, families are critical to help in these improvement processes; several tools that have been developed in recent years can be highlighted. In this sense, there are instruments regarding attention to diversity for a quality IE that are aimed at teachers, students, and families. In the case of families, concerning educational and social practices for inclusion, we distinguish the following existing instruments. The Attitudes Toward Inclusion Mainstreaming Scale [21]: this scale seeks to understand the attitudes of families with children with disabilities towards inclusion. Likewise, Domenech and Moliner [22] are the authors of the Questionnaire-Scale for Families on Inclusive Education, an instrument made up of three dimensions: knowledge, beliefs, and involvement of families in matters of IE. Benítez [23] designed the Questionnaire on the voice of parents of students with Down syndrome. It consists of a questionnaire that covers four dimensions: knowledge about integration and educational inclusion, organization of the school in terms of attention to diversity, relationship with the school, and general assessment of schooling.

Despite the importance of these questionnaires, one of the instruments, par excellence of the IE, is the Index for Inclusion [24]. The original work was published in 2000, and its adaptation to the Spanish environment was carried out in 2002 [25] with the denomination of “Guide for the evaluation and improvement of inclusive education” by the University Consortium for Inclusive Education. The next English version is from 2011, the latter was translated into Spanish in 2015 [24]. In addition, this questionnaire has been translated into more than 30 languages (French [26]; German [27]; Italian [28]; Portuguese [29]; Danish [30], among others). This instrument offers support to the process of self-review and alternative development of educational inclusion, as opposed to that based on inspection, competition, and fear of failure. In this sense, the index for inclusion provides an opportunity to develop an inclusive school in collaboration with others by stimulating individual and collective thinking, as well as the structure of the whole school and educational development of the community. It can be used by individual teachers, non-teachers, and parents/caregivers. It can lead to new dialogues about what children could learn in schools. The index for inclusion consists of two parts, one qualitative and one quantitative. The qualitative section focuses on fostering the process of reflection on a set of issues separated by domains (creating inclusive cultures producing inclusive policies and evolving inclusive practices) framed in terms of a set of activities. As for the quantitative section, the index for inclusion includes three questionnaires. The first one analyzes the answers of the students (63 items, unifactorial) and is intended to measure their perceptions about the extent to which the school, where they study, is inclusive. The second questionnaire analyzes answers of the family (56 items, unifactorial) and is intended to measure their perceptions about the extent to which the school, where their children study, is inclusive. Finally, the third questionnaire, related to teachers, is composed by 70 items across the factors, “Creating inclusive cultures” (21 items), “Producing inclusive policies” (22 items), and “Evolving inclusive practices” (27 items), and is intended to measure their perceptions about the extent to which the school where they work is inclusive. 

However, it should be noted that the Booth and Ainscow Index of Inclusion scale [31] has been used as a tool in studies from a qualitative point of view. In this sense, McMaster [32] carried out a study with 600 secondary school students, in which he used the Inclusion Index scale, specifically the qualitative part, to evaluate reflection processes, personal beliefs, and expectations in order to learn about the values of the school culture and reinterpret the educational policies that are being carried out in the educational institutions. On the other hand, Pillay et al., [33] carried out a study, using the qualitative part of the index for inclusion, focused on parents and teachers in several schools, concluding that awareness is needed for the educational context to integrate disability; it is important to involve the whole community in this process, infrastructure must be adapted, and institutional challenges overcome. Inclusive education must be promoted with the presence of all those involved (teachers, community, family members and people with disabilities). Similarly, Cruz-Ortiz, Pérez-Rodríguez, Jenaro-Rio, Sevilla-Santo, and Cruz-Ortiz [34] conducted a study with primary school students (69 without disabilities and 15 with disabilities) in which they used the quantitative part of the index for inclusion. This study demonstrated the relationship between inclusion and quality of life, as perceived by the participants. Neither the presence of Special Education Needs (SEN) nor the level of education seemed to influence the quality of life of the participants. These studies highlight that a fundamental aspect of improving inclusion in schools is based on understanding the nature of change and giving time to reflect on beliefs that may be deeply rooted. However, we believe that a quantitative perspective of the Inclusion Index Scale can offer relevant information to generate solid evidence when establishing statistical studies focused on the vision of the social context around the school, with respect to students with SEN.

### Objectives

The aim of the present study is to show evidence of validity and reliability of the Booth and Ainscow Index of Inclusion questionnaire to families. So far there is no evidence that this instrument has been quantitatively validated, despite having been used in multiple studies, which would give it more strength, efficacy, validity, and applicability to the results obtained, as well as more external validity (extrapolated to the rest of society). To this end, the aim of this work is to analyze the factorial structure of the questionnaire by means of a confirmatory factorial analysis, as well as analyze whether the construct measured through the instrument is invariant across genders. Finally, the reliability of the questionnaire was assessed.

## 2. Method

### 2.1. Participants

**Figure 1:**
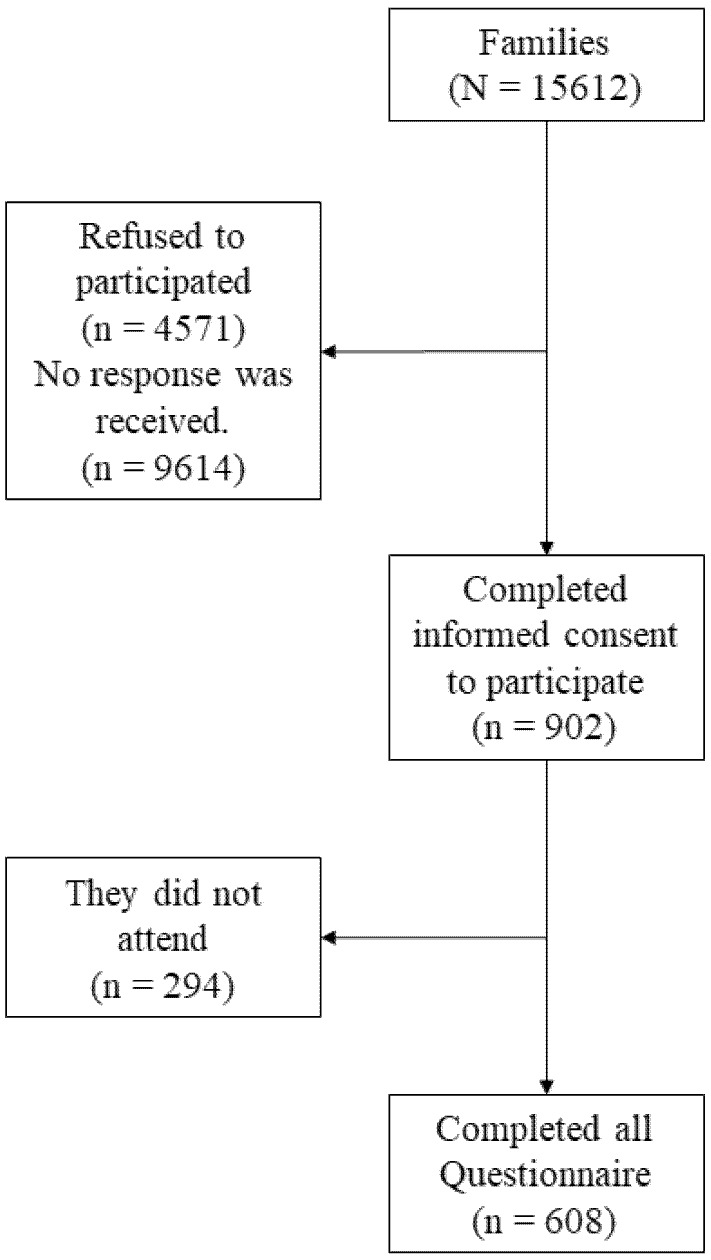
Sample flowchart. *N*: total; *n*: subtotal.

In this study, 108 fathers and 500 mothers took part (Figure 1), aged between 21 and 62 (*M* = 43.59; *SD* = 6.64), whose children belonged to non-university educational centers (private (37.34%) and public (62.66%)), specifically, compulsory secondary education (53.13%), vocational training (12.82%), infant and primary education (34.05%), distributed throughout Spain. 

The selection of the sample was non-probabilistic and incidental, based on the school centers we had access to and the families who wanted to participate. Likewise, the provision of informed consent was the criterion for participation in the study.

### 2.2. Measure

In this work we use the Spanish translation of the questionnaire “My child’s school”, a questionnaire included in the Index for Inclusion. The questionnaire is composed of 56 items organized into one single factor that measures the perceptions of families about the extent to which they consider—that the school where their children attend—offer them inclusive opportunities. The instrument is a self-report tool that is filled directly by respondents using a three-point Likert scale, where 0 = disagree, 1 = agree and disagree, 2 = agree.

### 2.3. Procedure

To carry out the study, we contacted the Federation of Associations of Parents of students, as well as the Association of Parents at a national level to ask for their collaboration, informing them of the objective of the research. For those parents who wanted to participate in the study, their consent was required, informing them beforehand of the objective of the study. Before administering the scale to all participants, it was completed by a small group of people to ensure correct understanding of all items. 

This research has been approved by the Bioethics Committee of the University of Almeria (Ref. UALBIO 2019/039). In addition, the ethical principles established by the American Psychological Association, and in accordance with the Declaration of Helsinki, were respected at all times. The estimated time to complete the questionnaire was around 15 minutes.

### 2.4. Data Analysis

To determine the validity and reliability of the questionnaire, a confirmatory factorial analysis (CFA) was first performed to test the factorial structure of the questionnaire. Once the definitive model was established, the present authors proceeded to the measurement and analysis of gender invariance and parental invariance by measuring equivalence and comparing the equality of the estimated parameters between the different groups. The procedure followed was that recommended by Byrne [35] and Kline [36], which consisted of evaluating the fit of a series of models in which restrictions were added compared to the base model (the configurable model in which no restrictions would be imposed). In this respect, a comparison was made between the unrestricted model (model 1) and three nested models (model 2 measurement weights, the factorial loadings were equal across groups; model 3 structural covariances, variances, and covariances in the structural part of the model were equal across groups; and model 4 measurement residues, variances, and covariances of the residual were equal across groups). Finally, the reliability of the instrument was evaluated through internal consistency analysis (Cronbach’s Alpha and Omega’s Coefficient). The statistical packages SPSS 25.0 (IBM, Armonk, NY, USA) and AMOS 20.0 (IBM, Armonk, NY, USA) were used for data analysis.

For the different CFAs, the maximum likelihood estimation method was used together with the bootstrapping procedure because the Mardia coefficient was high (189.87). The estimators were not affected by the lack of normality, so they were considered robust [35,36]. In order to accept or reject the models tested, a set of several fit indices was used: *χ*2/df, Comparative Fit Index (CFI), Incremental Fit Index (IFI), Root Mean Square Error of Approximation (RMSEA), plus 90% confidence interval (CI), and Standardized Root Mean Square Residual (SRMR). Since *χ*2 is very sensitive to sample size [37], *χ*2/df was used and values below 5 were considered acceptable [38]. Incremental indices (Comparative Fit Index, CFI; Tucker Lewis Index, TLI, and Incremental Fit Index, IFI) show a good fit with values equal to or greater than 0.90 [39], while error indices (RMSEA and SRMR) are considered acceptable with values equal to or less than 0.08 [40,41].

## 3. Results

### 3.1. Confirmatory Factorial Analysis

**Figure 2:**
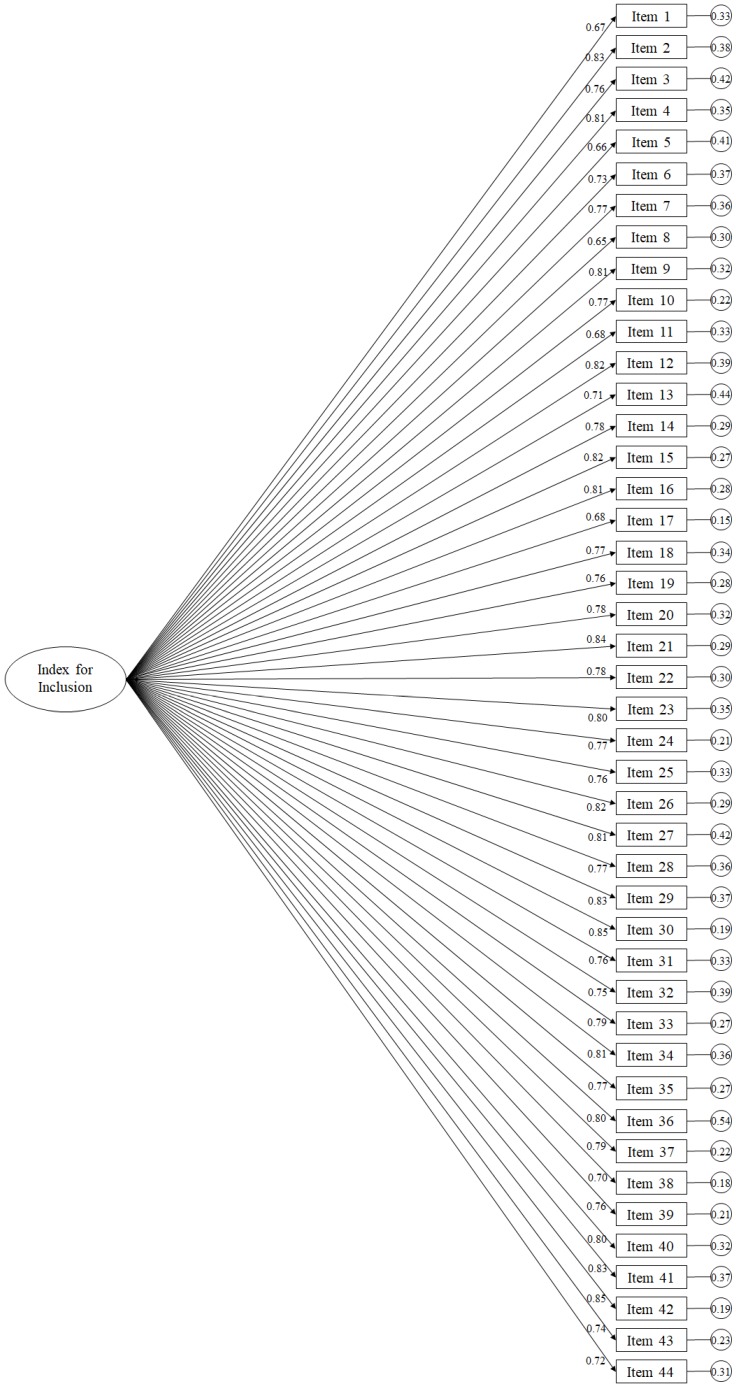
Confirmatory factor analysis of the questionnaire. The ellipses represent the factors and the rectangles represent the different items. The residual variances are shown in the small circles.

Initially, the structure of the 56-item, single-factor model was evaluated, with the following fit indices: *χ*2 (1484, *N* = 90) = 4124.69, *p* = 0.001; *χ*2/df = 3.78; CFI = 0.72; TLI = 0.71; IFI = 0.72; RMSEA = 0.082 (90% CI = 0.077–0.092); SRMR = 0.051. However, after analyzing the standardized regression weights, they ranged from 0.08 to 0.83 and were statistically significant (*p* < 0.001). Therefore, after observing these data, we proceeded to eliminate those items whose regression weights were less than 0.5, eliminating a total of 12 items [42]. Excluding these items, the model fit indices ostensibly improved: *χ*2 (902, *N* = 90) = 2553.78, *p* = 0.001; *χ*2/df = 2.85; CFI = 0.94; TLI = 0.94; IFI = 0.94; RMSEA = 0.064 (90% CI = 0.061–0.066); SRMR = 0.042. The final resulting model composed of 44 items (Figure 2), had statistically significant standardized regression weights (*p* < 0.001), ranging from 0.69 to 0.85.

### 3.2. Gender and Parent Invariance Analysis

The analyzed models can be considered as nested models in which constraints are progressively added. For the comparison of nested models, previous research used the maximum likelihood ratio test (*χ*2). The difference (Δ*χ*2) follows a *χ*2 distribution with degrees of freedom equal to the difference between the degrees of freedom (Δdf). If this value is statistically significant, in the comparison between the unrestricted model and model 2, it means that the restrictions specified in the more restrictive model are not sustained.

Table 1 and Table 2 show the various fit indices for the four models compared, respectively. In the case of the gender (Table 1) and parents (Table 2) models, no significant differences were found between model 1 (unrestricted) and model 2 (measurement weights). On the other hand, the results did reveal differences between model 1 and model 3 (structural covariances) and model 4 (measurement residuals). The absence of significant differences between model 1 and model 2 is a minimal criterion for accepting that the model structure is invariant with respect to gender and parents [43].

### 3.3. Descriptive Statistics and Internal Consistency Analysis

The mean scale score was 1.63 and the standard deviation was 0.26. To analyze the reliability of the scale (Table 3), an internal consistency analysis was carried out, which revealed a Cronbach alpha value of 0.92 for the parental inclusion index.

## 4. Discussion

The objective of this study was to show evidence of the validity of the Spanish version of the questionnaire that was referred to the parents of non-university education students, edited for Spain and Latin American countries, of the Booth and Ainscow Index for Inclusion questionnaire for parents [31], and translated into the Spanish context by Echeita, Muñoz, Simón, and Sandoval, starting with the English version of 2011. Until now, this questionnaire had been used qualitatively, in numerous studies, with the aim of supporting inclusion in educational centers through strategies of qualitative self-evaluation [44].

In the first place, the results of the present study revealed, through the CFA, the support to the factorial structure of the questionnaire formed by 44 items. This result was reached after analyzing the standardized regression weights, observing that these oscillated between 0.080 and 0.83, being statistically significant (*p* < 0.001). Therefore, after observing these data, we proceeded to eliminate the items where regression weights were less than 0.50, eliminating a total of 12 items. Subsequently, the questionnaire, made up of 44 items, had appropriate adjustment indices with regard to the analysis of invariance, with respect to gender, and parents of children with and without disabilities showing this invariance. In this way, parents of children with disabilities and parents of children without disabilities understood the questionnaire in a similar way. Therefore, future studies will be able to carry out comparative studies between different populations, taking into account sex, and parents of children with and without disabilities; thus, eliminating response bias on the part of the participants in the study, since both populations would understand it in a similar way. Next, two internal consistency analyses were performed, which revealed a Cronbach alpha value of 0.92 and an Omega’s Coefficient value of 0.81 for the parental inclusion index [45].

For all these reasons, this instrument can be of great value, as it makes it possible to better understand IE in schools from the perspective of parents of non-university education students and, especially, because it contributes to the construction of an inclusive society by promoting IE in schools. For these reasons, the future use of the Index for Inclusion for parents of non-university education students will be very interesting, as it will provide schools that are interested in the path toward IE additional information to that obtained through quantitative and reflection approaches, as the ones included in the first part of the Index for Inclusion. For example, it can make it possible to compare how parents from different schools included in a given neighborhood consider the degree of inclusion of their respective schools on the same metric. Although each school and school community are unique realities, the information provided through the use of this tool can give insight of the differences, to enrich collaboration of the schools and school improvement. In the first case, for example, parents who consider their child’s school as very inclusive can make initiatives to support the schools that have been assessed as less inclusive by other parents. On the other hand, if a school is seen as non-inclusive, this information can make authorities aware so as to provide a better distribution of resources, or to prioritize the implementation of initiatives to make the situation change. In this sense, a longitudinal study carried out by Alcaraz and Arnaiz [46] on special educational needs in Spain showed that, although the Spanish state has made great progress in the commitment toward inclusive education, the number of students with SEN enrolled in non-regular schools has increased in recent years. The conclusion is that it is necessary to promote policies for the schooling of students with SEN, which guarantee their presence in ordinary contexts in order to develop quality and inclusive educational care [47,48,49].

On the other hand, according to Vélez-Calvo, Tárraga Mínguez, Fernández Andrés, Pastor Cerezuela y Peñaherrera Vélez [50], inclusion must go beyond a strictly school environment and take shape as a social and community project. Therefore, inclusion must be worked on “from within” the school community itself, but with support and sustenance that “from outside”, from the state, autonomous and local entities, and should aim to achieve an inclusive education system. The Index is an extended tool to guide and orient towards inclusion. Its importance is based on reflection, willingness to improve, and the research attitude of the community students. Its use has not been limited to the school environment, but has been organized in a series of research papers that have verified the potential of this resource to guide the decisions that must be made in the implementation of inclusive education. Inclusion is the path and goal that schools follow in the search for education for all; research methodologies have been the way to illuminate this and identify the obstacles. 

### Limitations 

Although the results of the present study show consistent psychometric support, it is necessary to show some of the limitations. In the first place, this study has been developed with parents of students from educational centers throughout Spain, so it would be advisable to expand the sample size, extending the research to other Spanish speaking countries. Another limitation is the use of self-report measures, which, while appropriate for assessing subjective perceptions, could be supplemented in future research by other types of instruments and informants. Finally, in the present study, 12 items have been eliminated from the initial questionnaire, so in future studies it is recommended to analyze the internal factorial structure with our items and with all the items. 

## 5. Conclusions 

Sánchez [51] and Verdugo, Amor, Fernández, Navas, and Calvo [52] point out the need to develop valid and reliable standardized measures to support decision-making processes in relation to the inclusion of students with disabilities, especially when quantitative approaches are used, complying with the recommendations of the International Test Commission. Due to the relevance of this methodology, we have decided to apply it in the validation of our questionnaire. According to the results obtained, we can assert that the present questionnaire (Appendix A) has proved to be a robust instrument to assess inclusive education for parents of non-university education students, showing evidence of validity and reliability.

## Figures and Tables

**Table 1:** Multi-group analysis of gender invariance.

Model Index for Inclusion									
Models	*χ*2	*df*	*χ*2/*df*	Δ*χ*2	Δ*df*	CFI	IFI	RMSEA (IC 90%)	SRMR
Model 1	4492.12	1804	2.49			0.94	0.94	0.050 (0.048–0.051)	0.048
Model 2	4541.92	1847	2.46	49.80	43	0.94	0.94	0.049 (0.047–0.051)	0.048
Model 3	4542.88	1848	2.46	50.76	44 *	0.93	0.93	0.049 (0.047–0.051)	0.046
Model 4	4609.55	1892	2.44	117.42	88 **	0.93	0.93	0.049 (0.047–0.050)	0.046

* *p* < 0.05: ** *p* < 0.01. Note: model 1 = unrestricted; model 2 = measurement weights; model 3 = structural covariances; model 4 = measurement residuals; Comparative Fit Index (CFI); Incremental Fit Index (IFI); Root Mean Square Error of Approximation (RMSEA); Standardized Root Mean Square Residual (SRMR).

**Table 2:** Multi-group analysis of family invariance.

Model Index for Inclusion									
Models	*χ*2	*df*	*χ*2/*df*	Δ*χ*2	Δ*df*	CFI	IFI	RMSEA (IC 90%)	SRMR
Model 1	4482.10	1804	2.49			0.95	0.95	0.059 (0.056–0.061)	0.047
Model 2	4576.15	1847	2.48	94.05	43	0.95	0.95	0.058 (0.056–0.061)	0.047
Model 3	4636.67	1848	2.51	134.57	44 **	0.94	0.94	0.059 (0.057–0.061)	0.049
Model 4	4970.13	1892	2.63	288.03	88 **	0.94	0.94	0.061 (0.059–0.063)	0.049

** *p* < 0.01. Note: model 1 = unrestricted; model 2 = measurement weights; model 3 = structural covariances; model 4 = measurement residuals; Comparative Fit Index (CFI); Incremental Fit Index (IFI); Root Mean Square Error of Approximation (RMSEA); Standardized Root Mean Square Residual (SRMR).

**Table 3:** Reliability Statistics.

Cronbach’s Alpha	0.92
Omega’s Coefficient	0.81

## Appendix A

This instrument was validated in Spanish. It also includes the items in English.
1. Mi hijo por lo general quiere venir al centro escolar.
My child usually looks forward to coming to school.
2. Mi hijo tiene buenos amigos en el centro escolar.
My child has good friends at the school.
3. El centro escolar me mantiene bien informado sobre lo que ocurre en su interior.
The school keeps me well informed about what is going on.
4. Me han pedido que aporte en el transcurso de las lecciones con algún material o presencialmente en las clases.
I have been asked to make a contribution to lessons.
5. Creo que este es el mejor centro escolar de la zona.
I think this is the best school in the area.
6. Los estudiantes se llevan bien.
The children get on well together.
7. Los profesores se llevan bien.
The teachers get on well together.
8. Los adultos y estudiantes se llevan bien.
Adults and children get on well together.
9. Todas las familias son igualmente importantes para los profesores del centro escolar.
All families are equally important to the teachers at the school.
10. Tengo amigos entre los otros padres.
I have friends among the other parents.
11. Me gustan los profesores.
I like the teachers.
12. Los profesores se interesan sobre lo que le decimos acerca de mi hijo.
The teachers take an interest in what I tell them about my child.
13. Sólo por estar en el centro escolar mi hijo aprende a relacionarse con la gente.
Just by being at the school my child learns how to get on with people.
14. Mi hijo aprende lo que significa la democracia en el centro escolar.
My child learns what democracy means just by being at this school.
15. Mi hijo aprende sobre la importancia de cuidar el medio ambiente.
My child learns the importance of caring for the environment.
16. He estado involucrado en hacer del centro escolar un lugar mejor.
I have been involved in making the school a better place.
17. Cualquier niño que vive cerca de este centro escolar es bienvenido a venir aquí.
Any child who lives near to this school is welcome to come here.
18. Cada niño es tratado con respeto.
Every child is treated with respect.
19. Los chicos y chicas se llevan bien.
Boys and girls get on well together.
20. Ser gay o lesbiana es visto como una parte normal de la vida.
Being gay or lesbian or transgender is seen as an ordinary part of life.
21. Se es respetado independientemente del color de su piel.
You are respected irrespective of the colour of your skin.
22. Uno se considera parte del centro escolar, sea cualquiera su religión o si no tienen religión.
You are an equal part of the school whatever your religion or if you have no religion.
23. No se rechaza a los estudiantes debido a lo que llevan puesto.
People do not look down on children because of what they wear.
24. Se les respeta por su esfuerzo, no por los resultados obtenido en los exámenes.
You are respected for your effort not for the scores you get on tests.
25. Los estudiantes no se llaman con sobrenombres agresivos.
Children avoid calling each other hurtful names.
26. Si alguien acosa a mi hijo yo sé que recibiría ayuda del centro escolar.
If anyone bullied my child I know that I would get help from the school.
27. Si los estudiantes han estado fuera durante un día los profesores quieren saber dónde han estado.
If children have been away for a day a teacher wants to know where they have been.
28. Los profesores no tienen favoritos entre los estudiantes.
Teachers do not have favourites among the children.
29. Creo que los profesores son justos cuando elogian a un estudiante.
I think the teachers are fair when they praise a student.
30. Creo que los profesores son justos cuando castigan a un estudiante.
I think the teachers are fair when they punish a student.
31. Cuando los estudiantes están interrumpiendo las clases, otros estudiantes los calman.
When children are interrupting lessons other children help to calm them down.
32. Mi hijo aprende a resolver los desacuerdos escuchando, hablando y comprometiéndose.
My child learns how to settle disagreements by listening, talking and compromise.
33. Mi centro escolar envía a casa a los estudiantes que se han portado mal.
The school sends children home if they have behaved badly.
34. Las clases hacen buen uso de lo que mi hijo ha aprendido fuera del centro escolar.
Lessons make good use of what my child has learnt outside school.
35. Mi centro escolar tiene un buen sistema de apoyo a los estudiantes cuando tienen un problema.
The school has a good system for supporting children when they have a problem.
36. Mi hijo aprende mucho en este centro escolar.
My child learns a lot at this school.
37. A los estudiantes se les da confianza para aprender de forma autónoma.
Children are often trusted to learn on their own.
38. Mi hijo aprende a cuidar el medio ambiente en el centro escolar y su entorno.
My child learns to care for the environment in the school and the área around it.
39. Los estudiantes se ayudan unos a otros cuando están atascados en su trabajo.
The children help each other when they are stuck with their work.
40. Mi hijo sabe cómo obtener ayuda con su trabajo cuando es necesario.
My child knows how to get help with his or her work when it is needed.
41. El centro escolar es un lugar donde las personas realmente escuchan las ideas de otros.
The school is a place where people really listen to each other’s ideas.
42. Mi hijo siempre sabe lo que tiene que hacer después de que le expliquen la lección.
My child always understands what to do next in lesson.
43. Mi hijo suele entender qué tiene que hacer cuando le dan la tarea.
My child usually understands what to do when he or she is given homework.
44. Las tareas ayudan a mi hijo a aprender.
Homework helps my child to learn.
This questionnaire is measured by means of a Likert scale with three answer options:
0 = disagree
1 = agree and disagree
2 = agree
